# Supplementary material for: Spike Protein Cleavage-Activation in the Context of the SARS-CoV-2 P681R Mutation: an Analysis from Its First Appearance in Lineage A.23.1 Identified in Uganda
Source: Microbiol Spectr. 2022 Jun 29;10(4):e01514-22. doi: 10.1128/spectrum.01514-22 (PMC9430374; doi:10.1128/spectrum.01514-22)
Supplement: Supplemental file 1 — Fig. S1. Download spectrum.01514-22-s0001.pdf, PDF file, 0.1 MB [file spectrum.01514-22-s0001.pdf]

Supplementary Material

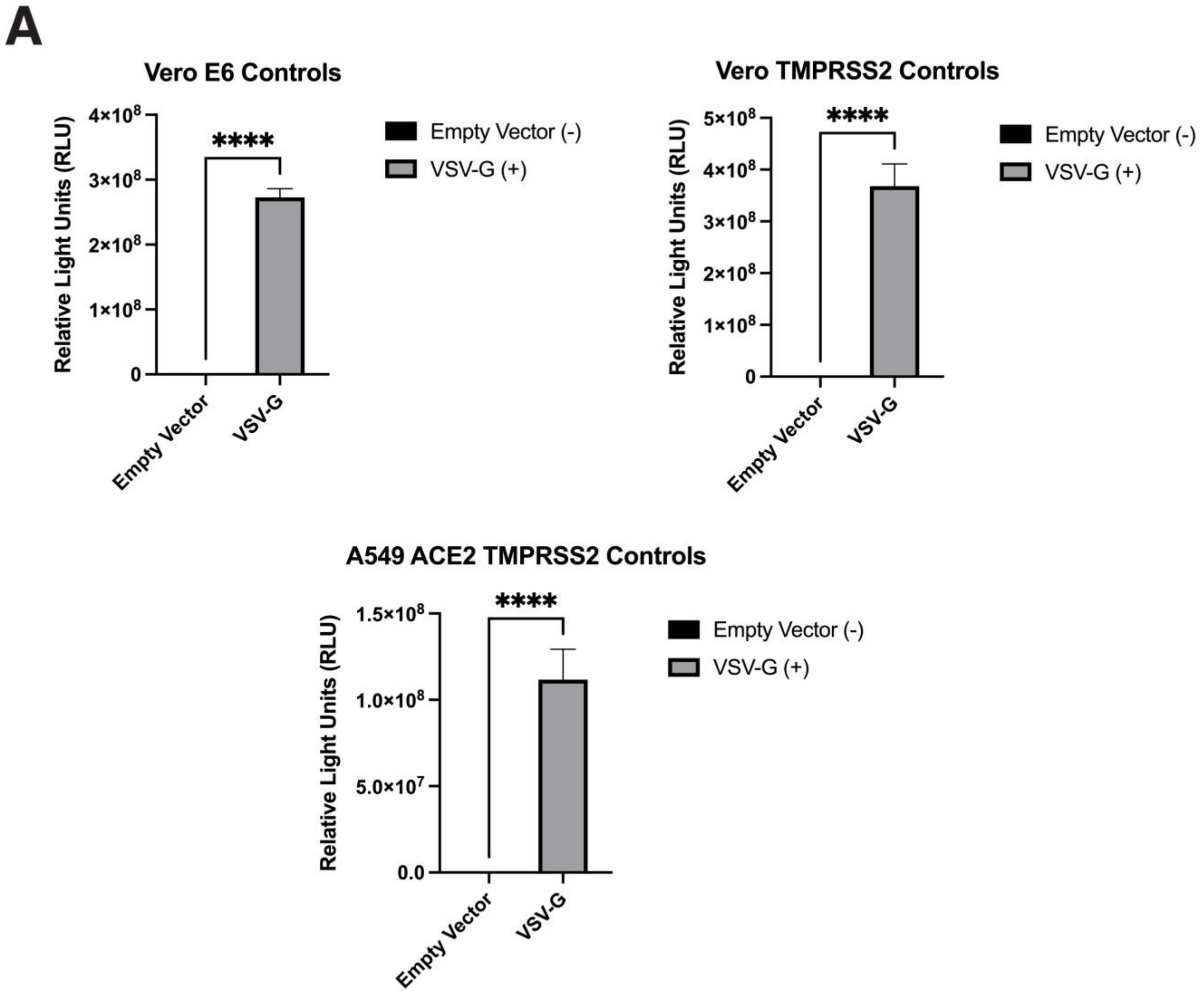

Supplementary Figure 1: MLV pseudoparticles controls. A. Pseudoparticle infectivity assays in Vero E6, Vero-TMPRSS2 and A549-ACE2-TMPRSS2 cells. Cells were infected with MLVpps harboring the VSV-G or no membrane glycoprotein (Empty vector). Data represents the average luciferase activity of cells of three independent experiments. Error bars represent G standard deviation (n = 3). Asterisks indicate statistical significance compared to the untreated control. Statistical analysis was performed using an unpaired Student's t test. **** $p < 0.0001$.
